# High Resolution Melting Analysis Targeting *hsp70* as a Fast and Efficient Method for the Discrimination of *Leishmania* Species

**DOI:** 10.1371/journal.pntd.0004485

**Published:** 2016-02-29

**Authors:** Ricardo Andrade Zampieri, Maria Fernanda Laranjeira-Silva, Sandra Marcia Muxel, Ana Carolina Stocco de Lima, Jeffrey Jon Shaw, Lucile Maria Floeter-Winter

**Affiliations:** 1 Physiology Department, Biosciences Institute, São Paulo University, São Paulo, São Paulo, Brazil; 2 Pathology Department, Medical Faculty, São Paulo University, São Paulo, São Paulo, Brazil; 3 Parasitology Department, Biomedical Institute, São Paulo University, São Paulo, São Paulo, Brazil; US Food and Drug Administration, UNITED STATES

## Abstract

**Background:**

Protozoan parasites of the genus *Leishmania* cause a large spectrum of clinical manifestations known as Leishmaniases. These diseases are increasingly important public health problems in many countries both within and outside endemic regions. Thus, an accurate differential diagnosis is extremely relevant for understanding epidemiological profiles and for the administration of the best therapeutic protocol.

**Methods/Principal Findings:**

Exploring the High Resolution Melting (HRM) dissociation profiles of two amplicons using real time polymerase chain reaction (real-time PCR) targeting heat-shock protein 70 coding gene (*hsp70*) revealed differences that allowed the discrimination of genomic DNA samples of eight *Leishmania* species found in the Americas, including *Leishmania (Leishmania) infantum chagasi*, *L*. *(L*.*) amazonensis*, *L*. *(L*.*) mexicana*, *L*. *(Viannia) lainsoni*, *L*. *(V*.*) braziliensis*, *L*. *(V*.*) guyanensis*, *L*. *(V*.*) naiffi* and *L*. *(V*.*) shawi*, and three species found in Eurasia and Africa, including *L*. *(L*.*) tropica*, *L*. *(L*.*) donovani* and *L*. *(L*.*) major*. In addition, we tested DNA samples obtained from standard promastigote culture, naturally infected phlebotomines, experimentally infected mice and clinical human samples to validate the proposed protocol.

**Conclusions/Significance:**

HRM analysis of *hsp70* amplicons is a fast and robust strategy that allowed for the detection and discrimination of all *Leishmania* species responsible for the Leishmaniases in Brazil and Eurasia/Africa with high sensitivity and accuracy. This method could detect less than one parasite per reaction, even in the presence of host DNA.

## Author Summary

The different clinical forms of the Leishmaniases range from cutaneous to visceral infections and are caused by organisms belonging to the genus *Leishmania*. Controversy over the validity of different molecular methods to correctly identify a species hinders the association of a given species with different clinical forms, complicating the prognosis and the development of suitable treatment protocols. A correct identification leads to a better understanding of the action and consequent development of new drugs and immunological reactions. It also provides important information about the relationship of each species with its hosts (humans, animal reservoirs and sandflies) in different geographical areas and ecological situations, helping to design control strategies. Today, PCR is the most commonly used method for *Leishmania* identification, but even though several targets have been described, no simple and direct protocol has emerged. In this paper, we coupled *hsp70* real-time PCR with the determination of amplicon melting profiles in order to explore polymorphic regions by HRM analysis. This methodology yielded discriminatory melting temperature (Tm) values for Brazilian and Eurasian/African *Leishmania* species. The protocol has proven to be 100% reliable with both clinical and experimental samples. The major advantage of the presently described method is that it is simple, less expensive, highly sensitive and easily automated.

## Introduction

Leishmaniases are a major worldwide public health problem and manifest themselves as a spectrum of diseases that may be exacerbated by other infections, such as human immunodeficiency virus. According to the World Health Organization, these diseases are endemic in 98 countries on 5 continents, with more than 350 million people at risk [1, 2]. Clinically, Leishmaniases can be broadly divided as either cutaneous or visceral, but neither form is exclusively linked to a particular species. Although cutaneous manifestations of the diseases are not life threatening, these manifestations can result in obstruction or destruction of the pharynx, larynx and nose in their final stages [2]. The visceral form is the most severe form, characterized by fever, loss of weight, splenomegaly, hepatomegaly, lymphadenopathies and anaemia, often with fatal outcomes if not timely treated [3].

The severity of the disease and its therapeutic responses are variable and depend on the patient’s immune response, the *Leishmania* species and even the parasite strain [4]. In this scenario, the development of optimized protocols for discriminating between the different *Leishmania* species is extremely useful and important in clinical management and treatment. The ability to evaluate the most appropriate species-specific treatments also supports the elucidation of the mechanisms of action of new drugs and the establishment of new species-specific treatment protocols. Furthermore, the identification of these parasites allows the generation of important data for clinical, epidemiological and ecological studies.

There are very few publications addressing a Leishmaniasis diagnosis using a High Resolution Melting (HRM) analysis, a methodology that detects differences in the nucleotide composition of a specific real-time PCR product. The method is based on thermodynamic differences in the dissociation curve profiles of amplicons generated from real-time PCR. The generated curves are specific signatures that identify polymorphisms due to small differences in nucleotide composition. In spite of the paucity of papers on the HRM method, some workers have already used it to discriminate *Leishmania* using targets against 7SL RNA [5, 6], *haspb* [7], the rRNA ITS sequence [8, 9], the rRNA ITS sequence coupled to *hsp70* [10, 11] and a FRET-based assay using MPI and 6PGD [12].

Amongst several targets described for *Leishmania* identification, the heat-shock protein 70 coding gene (*hsp70*) has proven to be useful in identifying many species of different geographical origins [13–17].

In this work, we propose a more efficient protocol using HRM analyses targeting the *hsp70* sequence for the discrimination of seven Brazilian *Leishmania* species, as well as three Eurasian and African species. This methodology was validated with DNA from reference strains, experimental infections in mice, human clinical samples and naturally infected phlebotomine sand flies.

## Materials and Methods

### Organisms

Promastigotes of *L*. *(L*.*) tropica* (MHOM/SU/60/OD), *L*. *(L*.*) donovani* (MHOM/IN/80/DD8), *L*. *(L*.*) infantum chagasi* (MCER/BR/1981/M6445), *L*. *(L*.*) major* (MHOM/IL/81/Friedlin), *L*. *(L*.*) amazonensis* (MHOM/BR/1973/M2269), *L*. *(L*.*) mexicana* (MNYC/BZ/62/M379), *L*. *(L*.*) lainsoni* (MHOM/BR/81/M6426), *L*. *(V*.*) braziliensis* (MHOM/BR/1975/M2903), *L*. *(V*.*) guyanensis* (MHOM/BR/1975/M4147), *L*. *(V*.*) naiffi* (MDAS/BR/1979/M5533) and *L*. *(V*.*) shawi* (MCEB/BR/84/M8408) were grown at 25°C in M199 medium with 10% fetal bovine serum (Life Technologies, Carlsbad, CA, USA). Procyclic forms of *Trypanosoma cruzi* (Y strain) and *T*. *brucei* (427 strain) were grown at 28°C in liver-infusion-tryptose medium and SDM-79, respectively, with 10% fetal bovine serum (Life Technologies). Human DNA, FMUSP-IOF-2016, obtained from USP Medical School, was used in specificity tests.

### Trypanosomatids DNA

DNA samples from reference strains were purified by a salting-out procedure using an adaptation of the protocol described by Miller et al. 1988 [18]. Approximately 2.5 x 10^9^ promastigotes in stationary growth culture were centrifuged at 3000 x g for 10 min at 25°C. The cells were resuspended in 6 mL of lysis buffer (10 mM Tris-HCl, pH 7.4; 400 mM NaCl; 2 mM EDTA) and lysed by the addition of 600 μL of 10% SDS. After overnight digestion with 1 mg of proteinase K at 37°C, 2 mL of saturated NaCl solution was added to lysate, and then, the lysate was vigorously mixed for 15 seconds and centrifuged for 15 minutes at 25°C for the removal of precipitated proteins. Two volumes of cold absolute ethanol were added to the supernatant, and the precipitated DNA was washed with 70% ethanol and resuspended in 1 mL of TE buffer (10 mM Tris, pH 7.4; 1 mM EDTA).

### Clinical, experimental and natural DNA samples/ethical statements

DNA from samples obtained from fresh humans biopsies, collected by doctors at Clinical Hospital of Medical Faculty USP, or fixed and paraffin-embedded samples from the collection of Instituto Evandro Chagas, (Belem-Para) were used in accordance to the norms established by the National Committee of Ethics in Research (Comissão Nacional de Ética em Pesquisa, CONEP/CNS), resolution 196/96 with the approval of the Ethics in Research Committees of the Institutions of origin (CAPPesq no. 0804/07, IEC n°. 0029/2007).

Fresh experimentally infected BALB/c mice samples of *L*. *(L*.*) amazonensis* or *L*. *(V*.*) braziliensis* were obtained 6 weeks after infection when the animals were sacrificed and tissues were collected and DNA was obtained as described below; the procedures involving the use of BALB/c mice had the approval of the Ethical Committee for use of Animals of Biomedical Sciences Institute of University of São Paulo (CEUA-ICB-USP), under protocol #145 of October 20^th^, 2011, according to Brazilian Federal Law 11.794 of October 8^th^ 2008.

DNA from infected phlebotomines captured in nature were purified using the commercial DNeasy Tissue & Blood kit (QIAGEN, Hilden, Germany), according to the manufacturer´s manual. Paraffin-embedded samples were prepared according to de Lima et al. 2011 [19]. The DNA concentration was measured by spectrophotometry.

### PCR assays

**Fig 1:**
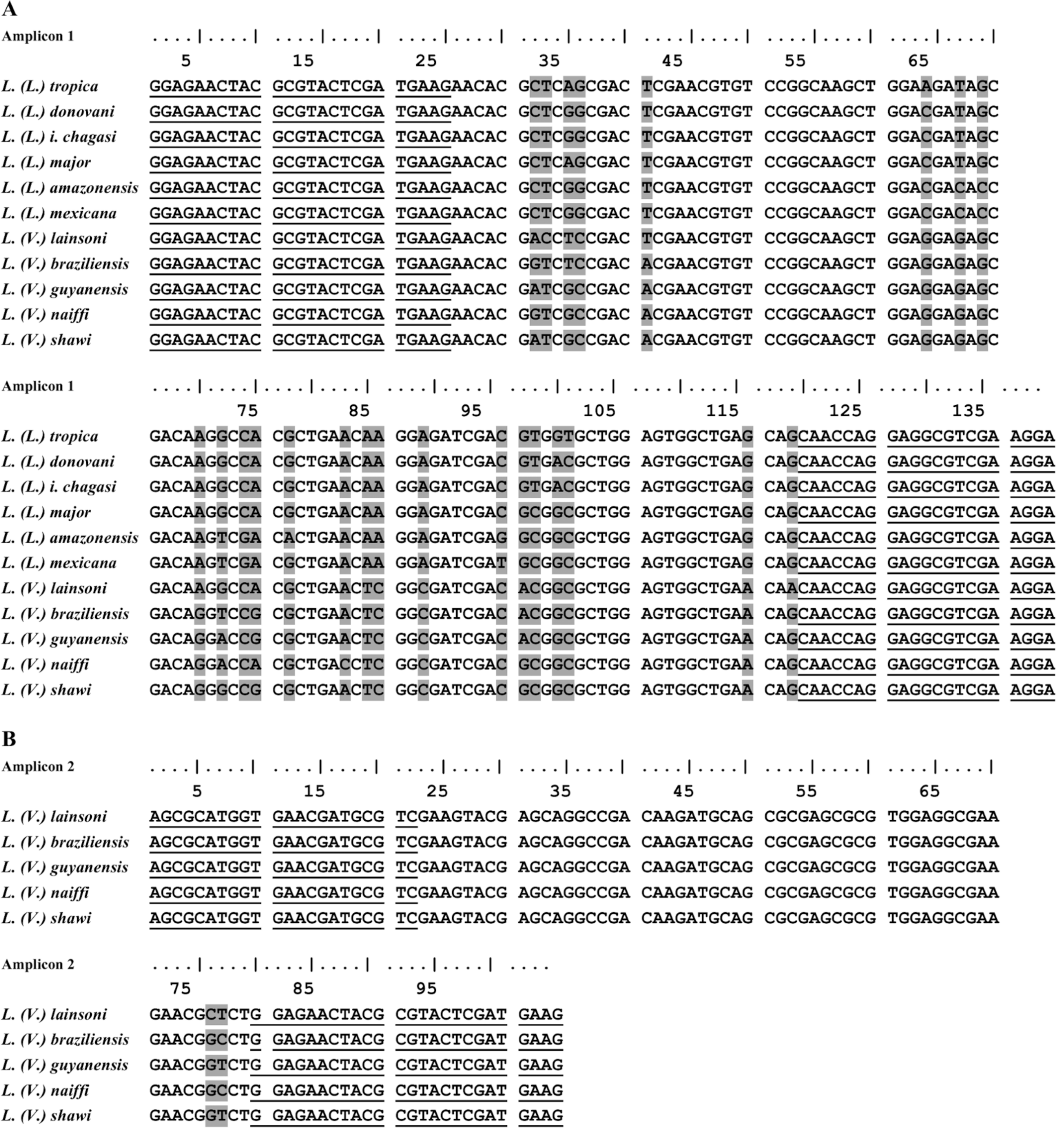
Nucleotide sequence of *hsp70* amplicons and primer localization. Alignment of the nucleotide sequence of amplicon 1 (A) and amplicon 2 (B) of each *Leishmania* species used in the HRM analysis. The underlined sequences indicate the position of the primers used in real-time PCR assays; the grey boxed nucleotides represent the variable regions found among reference strains of *Leishmania*.

Initially, we amplified the *hsp70* 234 bp fragments for all species analyzed in this study using the primers described by Graça et al. [17]. The alignment of the nucleotide sequence of those fragments was used to design primers for HRM analysis. Oligonucleotides used in the PCR assays to amplify a 144 bp fragment of *hsp70* (amplicon 1) were *hsp70*C reverse, previously described by Graça et al. 2012 [17], and a new forward oligonucleotide designed and named *hsp70*F2 (5’–GGAGAACTACGCGTACTCGATGAAG–3’). For the amplification of a 104 bp fragment of *hsp70* (amplicon 2) specific to the species from the *L*. *(Viannia)* subgenus, the oligonucleotides *hsp70*F1 (5’–AGCGCATGGTGAACGATGCGTC–3’) and *hsp70*R1 (5’–CTTCATCGAGTACGCGTAGTTCTCC–3’) were designed. The *hsp70* amplicon sequences are shown in Fig 1 and indicate the position of the primers. Conventional PCR reactions were performed on a Mastercycler Gradient Thermocycler (Eppendorf, Hamburg, Germany) with TopTaq Master Mix (QIAGEN) in a final volume of 25 μL with 200 nM of each primer and 50 ng of genomic DNA as a template. The thermal cycling conditions were as follows: an initial denaturation step of 94°C for 5 min, followed by 40 cycles of denaturation at 94°C for 1 min, annealing at 60°C for 30 sec and extension at 72°C for 30 sec, with a final extension at 72°C for 10 min. Real-time PCR reactions were performed using MeltDoc Master Mix for HRM with the fluorophore SYTO9 (Life Technologies) in a final volume of 20 μL with 200 nM of each primer and 50 ng of genomic DNA. The real time amplification conditions were as follows: an initial denaturation step at 94°C for 5 min, followed by 40 cycles of denaturation at 94°C for 30 sec and annealing/extension at 60°C for 30 sec, with the acquisition of fluorescent signals at the end of each extension step, followed by the dissociation curve for HRM analysis in Thermocycler PikoReal96 (Thermo Fisher Scientific, Walthman, MA, USA).

### Cloning and sequencing

The 234 bp *hsp70* fragment produced by conventional PCR, as described by Graça et al. 2012 [17], from each *Leishmania* species used in this study was purified and cloned into a pGEM-T vector using the pGEM-T Easy Vector System (Promega, Madison, WI, USA) and *E*. *coli* SURE competent cells. The recombinant plasmids from at least three colonies were purified, and they were sequenced with T7 and SP6 primers and the BigDyeTerminator v3.1 Cycle Sequencing Kit (Applied Biosystems, Foster City, CA, USA). The sequencing was performed on an ABI 3130 XL Platform (Life Technologies).

### Target quantification with standard curves

Recombinant plasmids containing the *hsp70* target were linearized with *Sca*I. The plasmid copy number was calculated considering the molar mass concentration, and a serial dilution on a tenth proportion was used to produce standard curves for each quantification test. The quality parameters for the standard curves were obtained by PikoReal Software (Thermo Fischer Scientific) analysis, including the correlation coefficient, linear dynamic range and PCR efficiency.

### High resolution melting (HRM)

HRM assays were performed at the end of each real-time PCR. The amplicon dissociation analysis was performed by capturing fluorescence signals in 0.2°C intervals and holding for 10 seconds in each range of the melting curve (between 60°C to 95°C). The acquisition of fluorescence data and the construction of dissociation profiles were performed using PikoReal96 software. HRM software normalizes melting curves relatively to values from pre- and post-melting point assigned as 100% and 0%, respectively. Then the software determines the normalized difference that means the signal-to-noise ratio difference of each sample versus a user-defined sequence that can be any. The call efficiency is the benchmark measured in percentage of the similarity between two dissociation profiles using fluorescence and Tm values as parameters. The software performs a paired comparison between the profile of the sample of unknown identity and each standard and chooses the standard that has the closest value. The “call” identity refers to the designation allotted to the sample being identified based on that of the closest standard.

The graphs containing the means and standard deviations of the Tm values obtained by the HRM analyses were made in GraphPad PRISM v. 6.02 software.

## Results

### Polymorphic sites on the hsp70 gene

The *hsp70* sequences deposited in GenBank for *L*. *(L*.*) tropica* (FN395025.1), *L*. *(L*.*) donovani* (AY702003.1), *L*. *(L*.*) infantum* (HF586351.1), *L*. *(L*.*) major* (HF586346.1), *L*. *(L*.*) amazonensis* (EU599090.1), *L*. *(L*.*) mexicana* (EU599091.1), *L*. *(L*.*) infantum chagasi* (FN395036.1), *L*. *(V*.*) braziliensis* (GU071173.1), *L*. *(V*.*) guyanensis* (EU599093.1), *L*. *(V*.*) lainsoni* (GU071174.1), *L*. *(V*.*) naiffi* (GU071183.1) and *L*. *(V*.*) shawi* (GU071177.1) were used for oligonucleotide design. DNA from all *Leishmania* reference strains analyzed in this study was used as templates in conventional PCR, and the amplicons were cloned and sequenced to confirm the sequences to those deposited in GenBank. The obtained *hsp70* amplicon sequences were then aligned, and we chose regions containing polymorphic sites to be used in HRM methodology (Fig 1).

The two pairs of oligonucleotides depicted in the alignment produced the two expected PCR fragments for all *Leishmania* reference strain DNA used as a template. The 144 bp amplicon 1 is the PCR product used in the amplification of all *Leishmania* species. The 104 bp amplicon 2 was produced by the oligonucleotide pair designed for species of the *L*. *(Viannia)* subgenus (Figs 1, S1 and S2).

### Specificity and sensitivity of HRM assays

**Fig 2:**
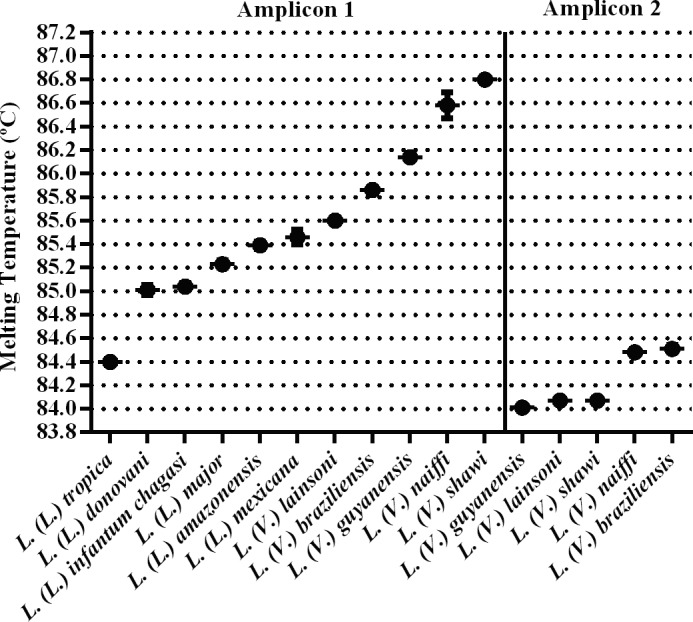
Tm values obtained with the HRM assay. Representative dispersion graph of individual Tm values for each studied *Leishmania* species using 50 ng of genomic DNA as a template for *hsp70* amplicon 1 and amplicon 2. The plots show the average and standard deviation of the Tm values. Each species was tested in duplicate in three independent experiments.

**Fig 3:**
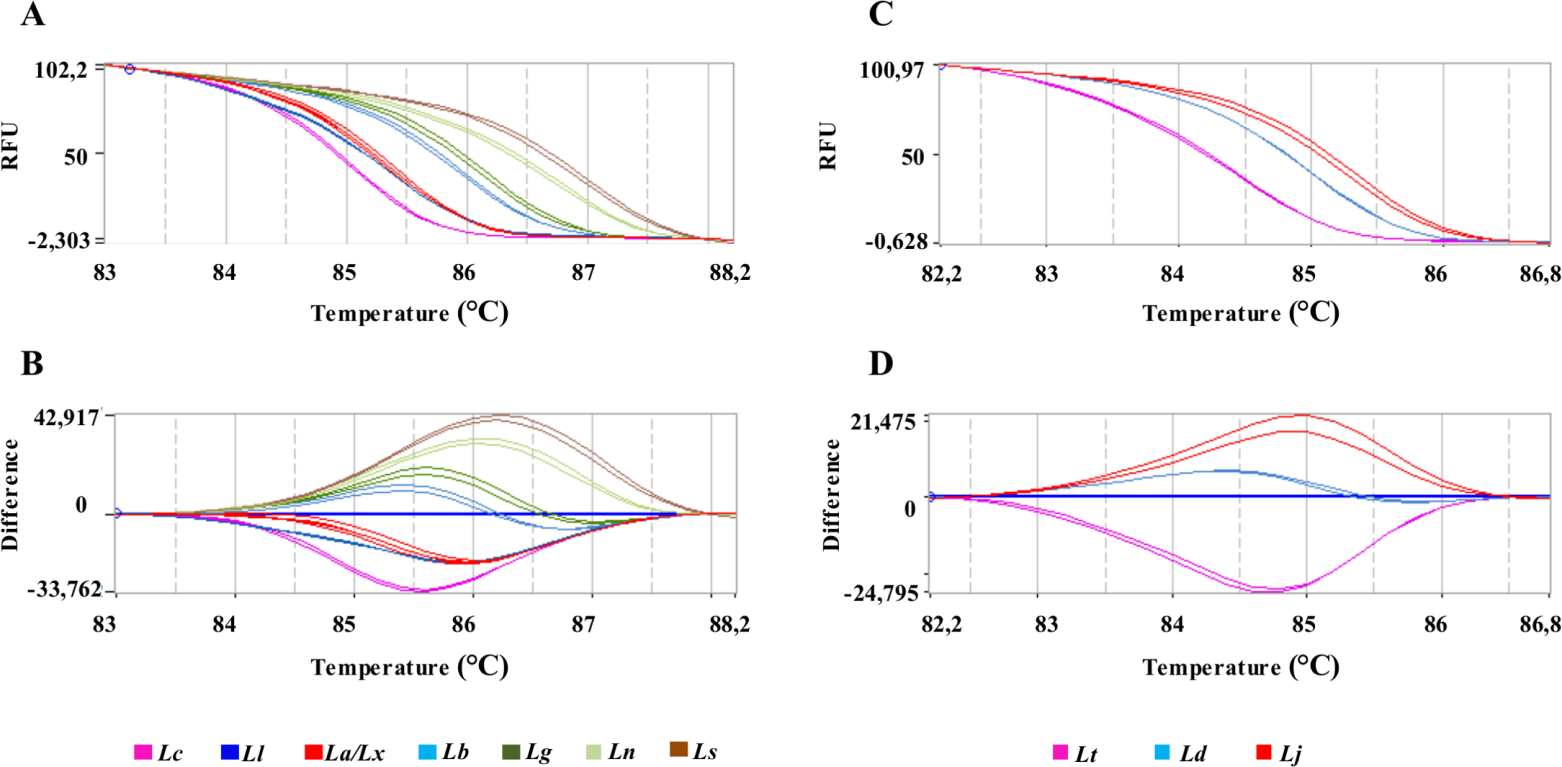
HRM plots of *hsp70* amplicon 1. Representative melting profiles of *hsp70* amplicon 1 obtained with DNA from *Leishmania* species present in Brazil (A, B) or DNA from *Leishmania* species of Eurasia and Africa (C, D). (A, C): Normalized melting curves; (C, D): normalized difference curves. (*Lt*): *L*. *(L*.*) tropica*; *(Ld*): *L*. *(L*.*) donovani*; (*Lc*): *L*. *(L*.*) infantum chagasi*; (*Lj*): *L*. *(L*.*) major*; (*La*): *L*. *(L*.*) amazonensis*; *(Lx*): *L*. *(L*.*) mexicana*; (*Ll*): *L*. *(V*.*) lainsoni*; (*Lb*): *L*. *(V*.*) braziliensis*; (*Lg*): *L*. *(V*.*) guyanensis*; (*Ln*): *L*. *(V*.*) naiffi* and (*Ls*): *L*. *(V*.*) shawi*. Each sample was tested in duplicate.

The average and standard deviation of the melting temperature (Tm) of each amplicon was determined in duplicate from three independent experiments using 50 ng of DNA as a template from each reference species. The melting profiles and obtained Tm values of *hsp70* amplicon 1 for all species studied are presented in Figs 2 and 3 and Table 1. For a reliable discrimination, we calculated the dispersion of Tm values and only considered differences in Tm values exceeding 0.3°C (Fig 2).

**Table 1:** Tm values obtained in the HRM analysis targeting the *hsp70* gene of different *Leishmania* species. Fifty ng of genomic DNA from each species was tested in duplicate in three independent experiments.

*Leishmania* species	Tm (°C) Amplicon 1	Tm (°C) Amplicon 2
*L*. *(L*.*) tropica*	84.40 ± 0.02	-
*L*. *(L*.*) donovani*	85.01 ± 0.04	-
*L*. *(L*.*) infantum chagasi*	85.04 ± 0.03	-
*L*. *(L*.*) major*	85.23 ± 0.03	-
*L*. *(L*.*) amazonensis*	85.39 ± 0.03	-
*L*. *(L*.*) mexicana*	85.46 ± 0.06	-
*L*. *(V*.*) lainsoni*	85.60 ± 0.01	84.07 ± 0.01
*L*. *(V*.*) braziliensis*	85.86 ± 0.01	84.51 ± 0.03
*L*. *(V*.*) guyanensis*	86.14 ± 0.02	84.01 ± 0.00
*L*. *(V*.*) naiffi*	86.58 ± 0.11	84.48 ± 0.01
*L*. *(V*.*) shawi*	86.80 ± 0.01	84.07 ± 0.01

The standard curves for the quantification assays using the cloned target showed good linear correlations (0.99 for all curves) and efficiencies varying from 92,37 to 97.23% for all tested species, in the range of 10^1^ to 10^7^ copies (S3 Fig). Moreover, to evaluate the specificity/sensitivity of *hsp70* amplicon 1 as a target, HRM assays were performed using genomic DNA from the seven references species of *Leishmania* in proportions of 1:1 or 1:100 in relation to a human reference DNA (FMUSP-IOF-2016), and the call identification agreed 100% with the reference samples, even in samples where the call efficiency was approximately 75% (Table 2).

**Table 2:** Call efficiency obtained in the HRM analysis targeting the *hsp70* gene of different *Leishmania* species in the presence of two different amounts of human reference (FMUSP-IOF-2016) DNA. The “call” column shows the identification name given by the software to the unknown samples based on names given to the reference samples; (CHA): *L*. *(L*.*) infantum chagasi*; (AMA): *L*. *(L*.*) amazonensis*; (LAI): *L*. *(V*.*) lainsoni*; (BRA): *L*. *(V*.*) braziliensis*; (GUY): *L*. *(V*.*) guyanensis*; (NAI): *L*. *(V*.*) naiffi* and (SHA): *L*. *(V*.*) shawi*. Each condition was tested in duplicate.

*Leishmania* species	DNA proportion 1:1		DNA proportion 1:100	
	Call	Efficiency (%)	Call	Efficiency (%)
*L*. *(L*.*) infantum chagasi*	CHA	92.55	CHA	79.95
*L*. *(L*.*) amazonensis*	AMA	89.90	AMA	75.80
*L*. *(V*.*) lainsoni*	LAI	89.20	LAI	74.35
*L*. *(V*.*) braziliensis*	BRA	89.85	BRA	78.10
*L*. *(V*.*) guyanensis*	GUY	91.90	GUY	79.40
*L*. *(V*.*) naiffi*	NAI	95.05	NAI	81.95
*L*. *(V*.*) shawi*	SHA	93.00	SHA	81.55

**Fig 4:**
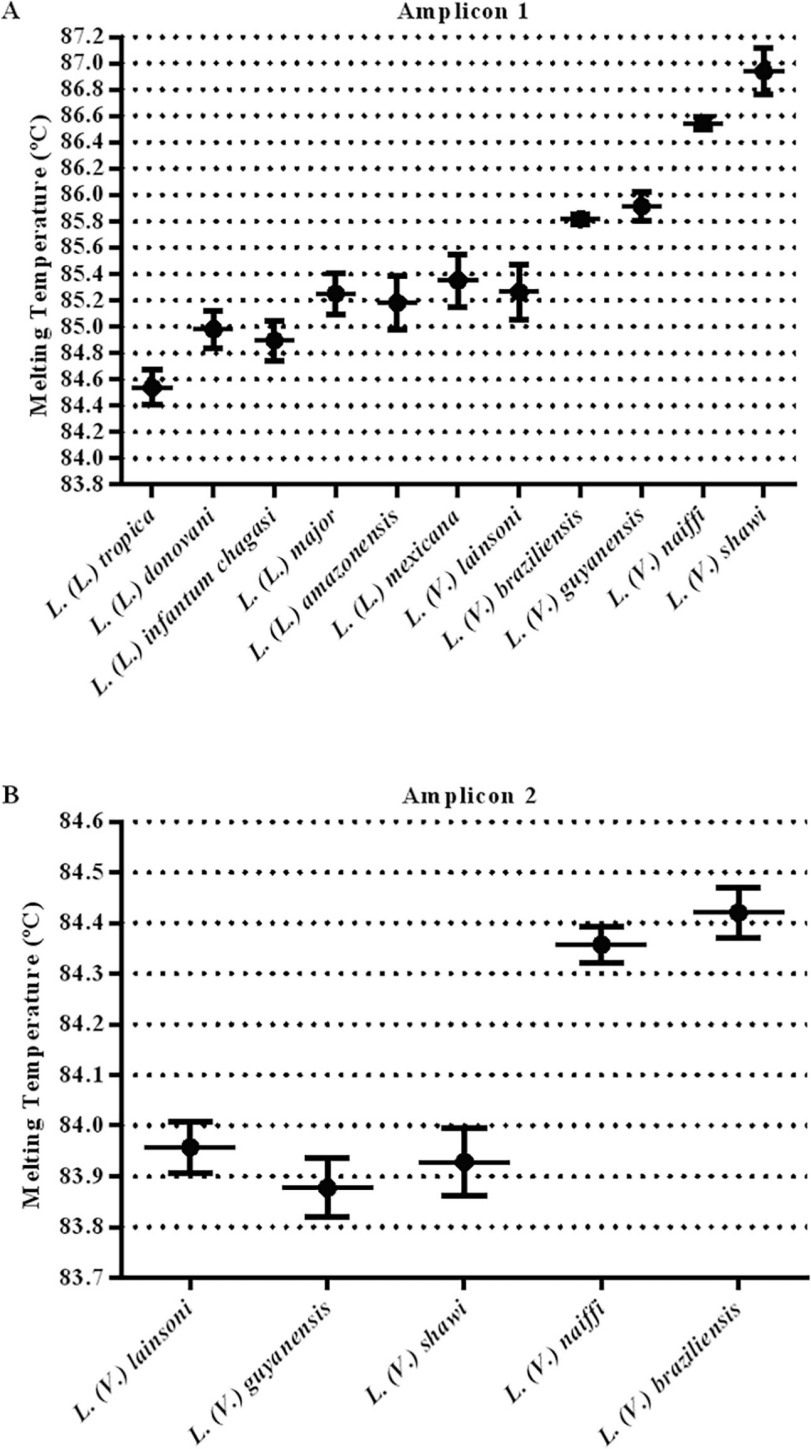
Effect of the amount of target DNA on the Tm values of *hsp70* amplicons. Representative dispersion graph of individual Tm values for each studied *Leishmania* species for amplicon 1 (A) and for amplicon 2 (B). Each point corresponds to the average and standard deviation of the variation in the Tms obtained within a range of 50 ng to 50 fg of genomic DNA used as a template. Each concentration point was measured in duplicate.

To test if the initial amount of target DNA caused a variation in the Tm, serial dilutions containing 50 ng to 50 fg (DNA amount corresponding to 5.0 x 10^5^ to 0.5 of parasite) of *Leishmania* DNA from reference strains were used as a template to produce both *hsp70* amplicon 1 (Fig 4A) and *hsp70* amplicon 2 (Fig 4B). The Tm variation obtained for both amplicons in each species showed that some species presented a fluctuation of Tm values that overlapped with other species.

**Fig 5:**
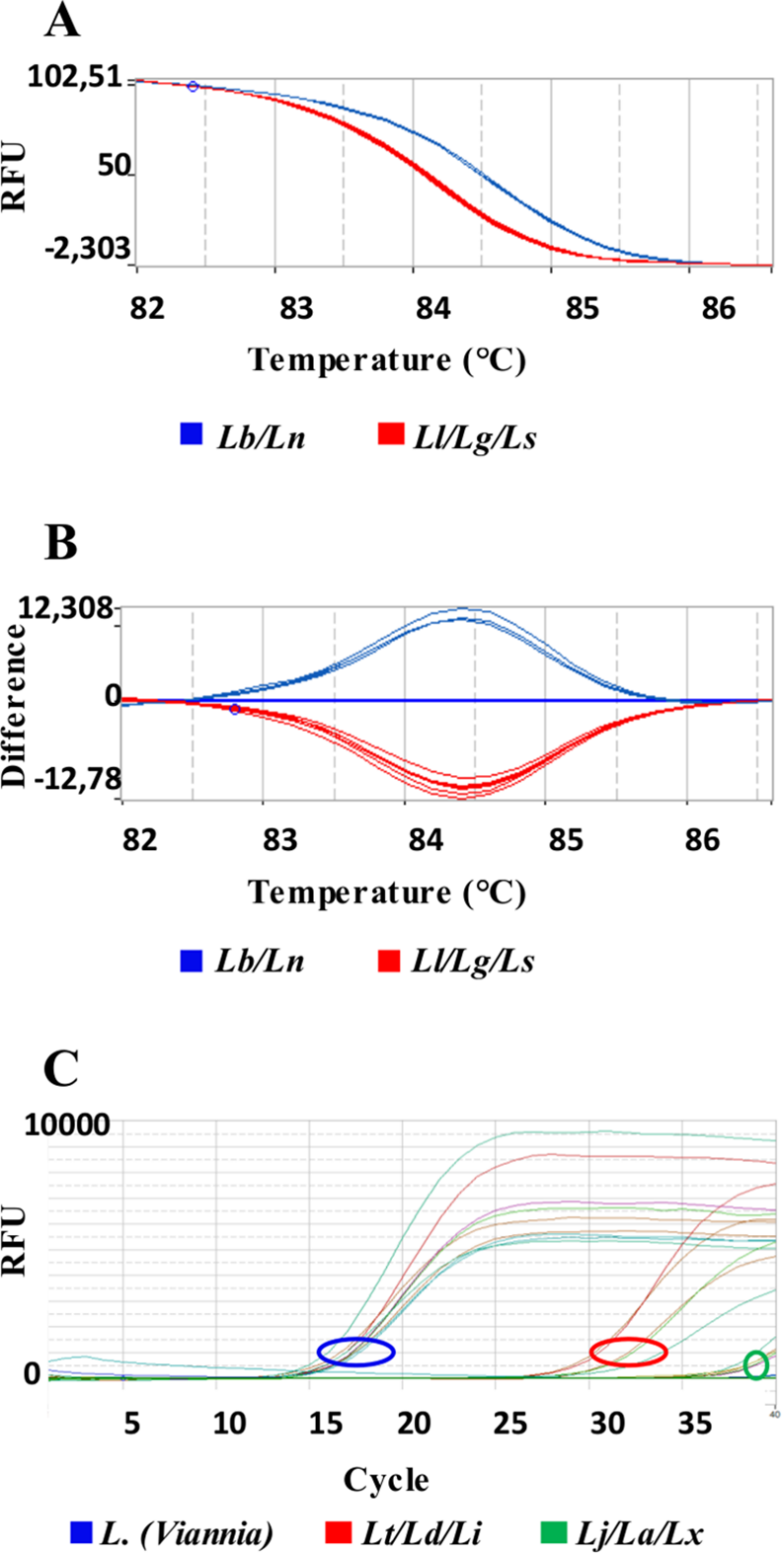
HRM plots of *hsp70* amplicon 2. Representative melting profiles of *hsp70* amplicon 2. Panels (A) and (B) show the melting profiles of American *L*. *(Viannia)* species with data organized in normalized melting curves and normalized difference curves, respectively. (*Lb*): *L*. *(V*.*) braziliensis;* (*Ln*): *L*. *(V*.*) naiffi*; (*Ll*): *L*. *(V*.*) lainsoni*; (*Lg*): *L*. *(V*.*) guyanensis* and (*Ls*): *L*. *(V*.*) shawi*. Panel (C) shows the amplification curves in relation to the Ct, using DNA of the same species as A and B plus *(Lt*.*)*: *L*. *(L*.*) tropica*; (*Ld)*: *L*. *(L*.*) donovani; (Li)*: *L*. *(L*.*) infantum; (La)*: *L*. *(L*.*) amazonensis; (Lx)*: *L*. *(L*.*) mexicana* and *(Lj)*: *L*. *(L*.*) major*. Each sample was tested in duplicate.

In the case of overlapping Tm values for amplicon 1, a sequential discrimination can be performed by HRM analysis of amplicon 2. This amplicon is specific for the *L*. *(Viannia)* subgenus species, allowing the segregation of two patterns that group *L*. *(V*.*) guyanensis*, *L*. *(V*.*) lainsoni* and *L*. *(V*.*) shawi* with Tm = 83.92 ± 0.04°C or *L*. *(V*.*) naiffi* and *L*. *(V*.*) braziliensis* with Tm = 84.39 ± 0.04°C (Figs 4 and 5).

The Ct values obtained in the amplification curves of amplicon 2, using DNA of all *Leishmania* studied indicated that the reactions were at least 5 orders of magnitude more specific to *Leishmania (Viannia)* species than for the *L*. *(Leishmania)* species (Figs 5C and S2), confirming that amplicon 2 can be used to discriminate *L*. *(Viannia)* from the *L*. *(Leishmania)* species.

Moreover, using the information on the geographical origin of the samples associated with the HRM analysis of *hsp70* amplicon 2 allowed for the discrimination between *L*. *(L*.*) donovani* and *L*. *(L*.*) infantum chagasi*; among *L*. *(L*.*) major*, *L*. *(L*.*) amazonensis*, *L*. *(L*.*) mexicana* and *L*. *(V*.*) lainsoni*.

DNA from uninfected mouse, human, or *Trypanosoma cruzi* and *T*. *brucei* were used as templates and compared to the standardized positive range of Tm values for the tested *Leishmania* species. No cross-reactivity was detected. For these controls, characteristic Tm values (*T*. *cruzi*: 83.08 ± 0.07°C and *T*. *brucei*: 83.91 ± 0.06°C) or no amplification was observed (mouse and human) (S4 Fig).

### Validation of the HRM Protocol with other Leishmania strains and with experimental, clinical and field samples

The HRM analysis of *hsp70* amplicon 1 obtained with DNA from other *Leishmania* isolates also used as reference strains resulted in a 100% correlation with the Tm values of the reference species used in this study (Table 3). Some of those strains represent isolates obtained from different geographical regions in Brazil, and experimentally corroborated the identification through the HRM protocol for possible polymorphisms.

The intra-specific variability was further assessed by the *in silico* analysis of polymorphism of 186 *hsp70* entries from *L*. *(L*.*) tropica*, *L*. *(L*.*) donovani*, *L*. *(L*.*) infantum*, *L*. *(L*.*) major*, *L*. *(L*.*) amazonensis*, *L*. *(L*.*) mexicana*, *L*. *(V*.*) lainsoni*, *L*. *(V*.*) braziliensis*, *L*. *(V*.*) guyanensis*, *L*. *(V*.*) naiffi*, *L*. *(V*.*) shawi*, *L*. *(V*.*) peruviana*, *L*. *(V*.*) panamensis*, *L*. *(L*.*) aethiopica*, *L*. *(L*.*) martiniquensis* and *L*. *siamensis*. All the sequences were aligned to include the regions of amplicons 1 and 2. The aligned sequences were then examined for polymorphisms among species as well as among strains of the same species. We then calculated the percentage of similarity and estimated the theoretical Tm value of both amplicons (S1 Table). If we assume that the nucleotide differences that we detected are real polymorphisms and not sequencing errors then we can see from S1 Table that the differences in the theoretical Tm values of each species results in the same discriminatory pattern. Of the 186 strains analyzed, only two strains of *L*. *infantum*, MCAN/IR/96/LON-49 and LEM75/zymodeme1, presented a theoretical Tm value whose difference was higher than 0.3°C. We cannot rule out the possibilities that this difference is in fact a real one, due to sequencing errors or reflects different taxa.

In the absence of *bona fide* samples we also determined the theoretical Tm of amplicons 1 and 2 (S1 Table) of two *Leishmania* species found in America, *L*. *(V*.*) peruviana* and *L*. *(V*.*) panamensis*, that occur outside Brazil. The obtained data indicated that these two species could be differentiated from the others *L*. *(Viannia)* species by the coupled HRM analysis of the two amplicons.

The theoretical Tm value of the African *L*. *(L*.*) aethiopica*, potentially allowed the discrimination from *L*. *(L*.*) donovani*, *L*. *(L*.*) infantum and L*. *(L*.*) major*, but not from *L*. *(L*.*) tropica* (S1 Table). The *enriettii* complex members *L*. *(L*.*) martiniquensis* and *L*. *siamensis* presented identical theoretical Tm values.

**Table 3:** Call efficiency obtained in the HRM analysis targeting the *hsp70* gene of different *Leishmania* strains The “call” column shows the names given by the software to the unknown samples based on names given to the reference samples: *L*. *(L*.*) infantum chagasi* (CHA), *L*. *(L*.*) amazonensis* (AMA), *L*. *(V*.*) lainsoni* (LAI), *L*. *(V*.*) braziliensis* (BRA), *L*. *(V*.*) guyanensis* (GUY), *L*. *(V*.*) naiffi* (NAI) and *L*. *(V*.*) shawi* (SHA). Each condition was tested in duplicate in three independent experiments.

*Leishmania* strain / ID	Call	Efficiency (%)
*L*. *(L*.*) infantum chagasi* / MHOM/BR/74/M2682	CHA	67.88
*L*. *(L*.*) amazonensis* / MPHI/BR/99/M12275	AMA	83.35
*L*. *(V*.*) lainsoni* / IUBI/BR/91/M13469	LAI	73.72
*L*. *(V*.*) braziliensis* / MHOM/BR/2001/M19675	BRA	85.47
*L*. *(V*.*) braziliensis* / MHOM/BR/96/M15923	BRA	81.12
*L*. *(V*.*) guyanensis* / MHOM/BR/2001/M19869	GUY	78.73
*L*. *(V*.*) naiffi* / MDAS/BR/82/M6934	NAI	87.75
*L*. *(V*.*)* shawi / MHOM/BR/2001/M19670	SHA	91.10
*L*. *(V*.*) shawi* / MHOM/BR/2001/M19664	SHA	87.90
*L*. *(V*.*) shawi* / MHOM/BR/1990/M19703	SHA	88.10

To validate the HRM protocol for different types of sample preparations, sixteen DNA obtained from real biological samples, like fresh tissue from hamster inoculated with infected sample from human or dog cases; cell culture of the human isolated strain; human fresh tissue; human paraffin-embedded tissue; tissues from experimentally infected BALB/c mice and naturally infected phlebotomines, that had been previously tested in our laboratory by sequencing of SSU rDNA [20] or by discriminatory PCR targeting *g6pd* [21], were submitted to HRM analysis. The results obtained presented a correlation with the results obtained with the other targets (Table 4).

**Table 4:** Identification of *Leishmania* in clinical and experimentally infected and field samples by the HRM analysis targeting *hsp70* gene. The *hsp70* amplicons 1 or 2 of DNA from each sample were submitted to the HRM analysis. The results were compared with a previous identification performed by SSU rDNA sequencing [17] or *g6pd* PCR [18]. ^(a)^ fresh tissue from hamster inoculated with infected sample; ^(b)^ cell culture of the human isolated strain; ^(c)^ human fresh tissue; ^(d)^ human paraffin-embedded tissue; ^(e)^ experimentally infected BALB/c mice; ^(f)^ naturally infected *Lutzomyia (Lutzomyia) longipalpis*; and ^(g)^ naturally infected *Lu*. *(Nyssomyia) whitmani*; (N/A): not applicable.

Sample Source	HRM Identification		Previous Diagnosis	Method
	Amplicon 1	Amplicon 2		
Human^**a**^	*L*. *(V*.*) braziliensis*	*L*. *(V*.*) braziliensis*	*L*. *(Viannia)* sp.	SSU + seq
Human^**a**^	*L*. *(V*.*) braziliensis*	*L*. *(V*.*) braziliensis*	*L*. *(Viannia)* sp.	SSU + seq
Human^**a**^	*L*. *(V*.*) braziliensis*	*L*. *(V*.*) braziliensis*	*L*. *(Viannia)* sp.	SSU + seq
Human^**a**^	*L*. *(V*.*) braziliensis*	*L*. *(V*.*) braziliensis*	*L*. *(V*.*) braziliensis*	*g6pd* PCR
Human^**b**^	*L*. *(V*.*) braziliensis*	*L*. *(V*.*) braziliensis*	*L*. *(V*.*) braziliensis*	*g6pd* PCR
Human^**b**^	*L*. *(V*.*) braziliensis*	*L*. *(V*.*) braziliensis*	*L*. *(V*.*) braziliensis*	*g6pd* PCR
Human^**b**^	*L*. *(V*.*) braziliensis*	*L*. *(V*.*) braziliensis*	*L*. *(V*.*) braziliensis*	*g6pd* PCR
Human^**c**^	*L*. *(V*.*) braziliensis*	*L*. *(V*.*) braziliensis*	*L*. *(V*.*) braziliensis*	*g6pd* PCR
Human^**d**^	*L*. *(V*.*) braziliensis*	*L*. *(V*.*) braziliensis*	*L*. *(Viannia)* sp.	SSU + seq
Canine^**a**^	*L*. *(V*.*) braziliensis*	*L*. *(V*.*) braziliensis*	*L*. *(Viannia)* sp.	SSU + seq
Canine^**a**^	*L*. *(L*.*) infantum chagasi*	N/A	*L*. *(L*.*) infantum chagasi*	SSU + seq
Mouse^**e**^	*L*. *(L*.*) amazonensis*	N/A	*L*. *(L*.*) amazonensis*	SSU + seq
Mouse^**e**^	*L*. *(L*.*) amazonensis*	N/A	*L*. *(L*.*) amazonensis*	SSU + seq
Mouse^**e**^	*L*. *(V*.*) braziliensis*	*L*. *(V*.*) braziliensis*	*L*. *(V*.*) braziliensis*	*g6pd* PCR
Phlebotomine^**f**^	*L*. *(L*.*) infantum chagasi*	N/A	*L*. *(L*.*) infantum chagasi*	SSU + seq
Phlebotomine^**g**^	*L*. *(V*.*) braziliensis*	*L*. *(V*.*) braziliensis*	*L*. *(V*.*) braziliensis*	*g6pd* PCR

## Discussion

The establishment of optimized protocols for the detection and identification of the aetiological agents of Leishmaniases are extremely useful tools in a clinical context. Identifying the species can lead to species-specific treatment protocols to promote a better efficacy of treatment, assessing the need for patient follow up as well as the development and understanding of the mode of action of potential new drugs.

Several methodologies targeting different genomic or mitochondrial DNA have been described in the past 20 years, and PCR is currently the preferred method in studies involving the detection and identification of *Leishmania*. These methodologies have been developed by designing primers that exploit species-specific sequence polymorphisms in different targets, such as kDNA [22], the SSU rDNA gene [20, 23], the glucose-6-phosphate dehydrogenase gene (*g6pd*) [21, 24], rDNA internal transcribed spacers (ITSs) [25], *hsp70* [13–17] and cysteine proteinase B gene (*cpb*) [7, 26]. However, none of these methods represents a gold standard because the targeted polymorphisms were unsuitable for simple and direct identification protocols. These PCR analyses involved the use of multiple targets requiring a combination of several primers creating the need of running more than one reaction to identify a single sample. The multiplex PCR that uses several pair of primers in one reaction and restriction fragment length polymorphism analysis (RFLP) of PCR products both need of a subsequent DNA fractionation by gel electrophoresis. These procedures require experienced operators to interpret the results, besides the risk of laboratory contamination with amplicons, due to the manipulation of PCR product.

Another way to exploit DNA polymorphisms is the determination of the C+G composition of PCR products from conserved regions by calculating the Tm of the amplicon in a melting curve. HRM methodology has been successfully used for *Leishmania* identification using different targets, such as the 7SL RNA gene that discriminated *L*. *tropica*, *L*. *major* and species that cause visceral *Leishmania*ses in clinical samples [5, 6]. Additionally, using the same target, researchers determined that rodent *Ctenodactylus gundi* is a potential host of *L*. *tropica* in Tunisia [5]. Polymorphisms on *haspb* (Hydrophilic Acylated Surface Protein B gene) analyzed by HRM allowed the differentiation of strains of *L*. *(L*.*) donovani* from distinct regions of East Africa [7]. In Southeastern Iran, the rRNA ITS sequence incriminated *Phebotomus sergenti* as a natural vector of *L*. *(L*.*) tropica* [10], or the discrimination between *L*. *(L*.*) tropica* and *L*. *(L*.*) infantum* in Turkey [9]. HRM analysis of the ITS-1 rRNA region discriminated *L*. *(L*.*) major*, *L*. *(*.*L) tropica*, *L*. *(L*.*) aethiopica* and *L*. *(L*.*) infantum* in samples from Middle East, Asia, Africa and Europe [8]. The combination of two targets, *hsp70* and the rRNA ITS1 sequence, using the absolute HRM values allowed for the discrimination of six American *Leishmania* species [11] and MPI/6PGD-FRET PCR distinguished *L*. *(V*.*) braziliensis* from *L*. *(V*.*) peruviana* [12].

**Fig 6:**
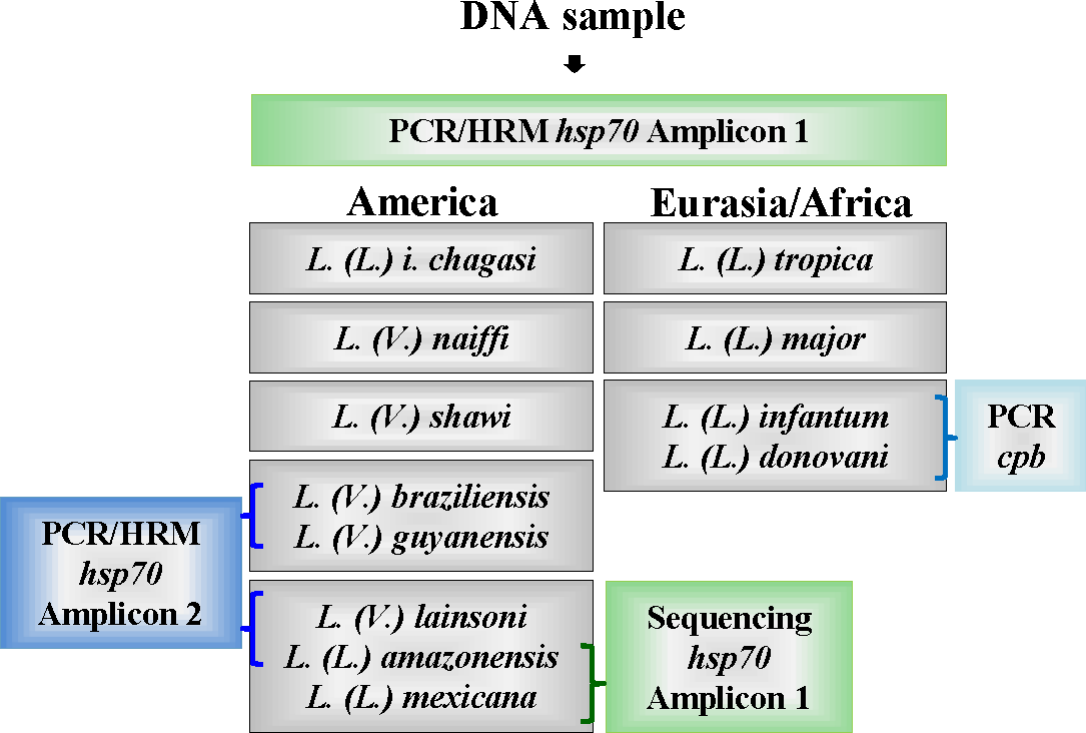
Schematic algorithm representation for *Leishmania* identification in Brazilian or Eurasian/African samples. Purified DNA was submitted to *hsp70* PCR to produce amplicon 1. The melting analysis of *hsp70* amplicon 1 for American samples discriminates *L*. *(L*.*) infantum chagasi*, *L*. *(V*.*) naiffi* and *L*. *(V*.*) shawi*. The grouping of *L*. *(V*.*) braziliensis/L*. *(V*.*) guyanensis* can be discriminated by producing *hsp70 PCR* amplicon 2. Amplicon 2 also resolve the grouping *L*. *(L*.*) amazonensis/L*. *(L*.*) mexicana/L*. *(V*.*) lainsoni*, which is positive for *L*. *(V*.*) lainsoni*, or by sequencing *hsp70* PCR amplicon 1 to discriminate between *L*. *(L*.*) amazonensis and L*. *(L*.*) mexicana*. For Eurasian/African samples, the melting analysis of *hsp70* amplicon 1 discriminates *L*. *(L*.*) tropica*, *L*. *(L*.*) major* and the *L*. *(L*.*) donovani/L (L*.*) infantum* group, which can be solved by *cpb* PCR [7].

Here, we described an algorithm using HRM methodology for the rapid detection and discrimination of *Leishmania* species circulating in Brazil and Eurasia/Africa (Fig 6). We used the sequence coding for *hsp70*, but in order to obtain a discriminatory PCR product, we designed the primers to encompass a region that was no larger than 144 bp and that had relevant polymorphisms for HRM analysis, that is, shifts of AT base pairs to CG or vice-versa. Moreover, to be effective, the total amount of polymorphisms was taken into account, and compensatory changes were avoided. Using these criteria, we obtained two PCR products: amplicon 1 and amplicon 2. Using the algorithm described in Fig 6, the analysis of the produced melting profiles of amplicon 1 for the Brazilian species allowed for the discrimination of *L*. *(L*.*) i*. *chagasi*, *L*. *(L*.*) amazonensis/L*. *(L*.*) mexicana/L*. *(V*.*) lainsoni*, *L*. *(V*.*) braziliensis/L (V*.*) guyanensis*, *L*. *(V*.*) naiffi* and *L*. *(V*.*) shawi* using differences in the Tm of at least 0.3°C. For Eurasian samples, amplicon 1 produced values with the same 0.3°C interval to discriminate *L*. *(L*.*) tropica* from *L*. *(L*.*) major* and from *L*. *(L*.*) donovani/L (L*.*) infantum*, but these two species cannot be discriminated from each other (Fig 2).

The occurrence of an overlap in the Tm value for the Brazilian species *L*. *(L*.*) amazonensis* and *L*. *(L*.*) lainsoni* after a positive reaction of amplicon 1 can be solved by a positive reaction of amplicon 2. This amplicon sequence is specific for *Leishmania (Viannia)* species, so *L*. *(L*.*) amazonensis* will not be amplified and *L*. *(V*.*) lainsoni* will present the corresponding Tm value (Fig 5). The occurrence of an overlap in the Tm value for the American species *L*. *(L*.*) amazonensis* and *L*. *(L*.*) mexicana* can be solved by amplicon 1 sequencing because this amplicon is not identical, but there are two mismatches (position 82 A to G and position 100 G to T in *L*. *(L*.*) amazonensis* and *L*. *(L*.*) mexicana*, respectively (Fig 1), that are compensatory in the melting profile. It is interesting to note that these two species are very closely related. Uliana et al. [23] distinguished *L*. *(L*.*) amazonensis* from *L*. *(L*.*) mexicana* by SSU rDNA, but Castilho et al. [21] also failed to distinguish these species by *g6pd* because the region of the *g6pd* sequence that was used is identical in the two species. It is also interesting that Hernandez et al. [11], using a larger amplicon (337 bp) of *hsp70*, succeeded in differentiating *L*. *(L*.*) mexicana* from *L*. *(L*.*) amazonensis*; however, Fraga et al. [13] failed to distinguish these two species using RFLP in another region of *hsp70*. However, when the complete nucleotide sequence of the *hsp70* PCR fragment of 1268 bp is used, the discrimination between the two species can be achieved [27]. These problems once again emphasize that one gene or a particular sequence of a gene is not reliable to define a species or plot its phylogeny. Recently, Real et al. [28] showed that *L*. *(L*.*) mexicana* and *L*. *(L*.*) major* had, respectively, 5 and 7 species-specific orthologous gene families, while *L*. *(L*.*) amazonensis* had 23 different gene families. Moreover, the geographical parameter can also be used; Uliana et al. used SSU rDNA polymorphism to show that these species present a characteristic distribution in Latin America that correlates to monoclonal antibody profiles [29].

The Tm overlap for Eurasian species occurred for *L*. *(L*.*) donovani* and *L*. *(L*.*) infantum*, which presented identical sequences for amplicon 1. Again, the geographical origin of the sample can be used because *L*. *(L*.*) donovani* is more frequently found in India and East Africa and presents anthroponotic behavior. *L*. *(L*.*) infantum* is found in Africa, China and the Mediterranean and shows zoonotic behavior [30]. However, the two species can be discriminated by multilocus enzyme electrophoresis (MLEE) or multilocus microsatellite typing (MLMT) [30]. Recently, the *haspb* coding region was initially used in a classical PCR coupled to RFLP [31], while the gene coding for *cpb* was used as a target in conventional PCR [7]. We propose to use the latter in case of doubt between the two species (Fig 6).

The *in silico* analysis of amplicon 1 and 2 from other *Leishmania* species from America or from Eurasia/Africa, also indicated the potentiality of the *hsp70* HRM protocol to discriminate *L*. *(V*.*) peruviana*, *L*. *(V*.*) panamensis* and *L*. *(L*.*) aethiopica/ L*. *(L*.*) martiniquensis*/*L*. *siamensis* from *L*. *(L*.*) donovani* and *L*. *(L*.*) major* but not from *L*. *(L*.*) tropica*. It is interesting to note that the ITS-HRM analysis applied to *L*. *(L*.*) tropica L*. *(L*.*) aethiopica*, *L*. *(L*.*) infantum*, *L*. *(L*.*) major* and *L*. *(L*.*) donovani* [8] presented exactly the same degree of resolution of the *hsp70* HRM described here.

We also noticed that the initial amount of template DNA influenced the Tm determination (Fig 4). This Tm variation could be important in cases where the Tm values are in the same range and can lead to a misidentification if the reference sample is at a different concentration. This is the case for *L*. *(L*.*) amazonensis* and *L*. *(L*.*) lainsoni*. However, as has been previously explained, the use of *hsp70* amplicon 2 allowed for the discrimination between these two species. The two other species that presented an overlapping Tm range depending on the initial amount of DNA were *L*. *(V*.*) braziliensis* and *L*. *(V*.*) guyanensis*, which could be discriminated by the use of an HRM analysis on the same amplicon 2.

In fact, when we applied the protocol described here to other *Leishmania* isolates, the obtained “call” (the identification of the problem sample in relation to the reference samples) presented a 100% correlation with the reference strains (Table 3).

The test of sixteen samples consisting of fresh hamster tissue from animals injected with human or dog biopsy macerates, fresh or paraffin embedded human biopsies, tissues of experimentally infected BALB/c mice or even naturally infected phebotominae, produced identification “calls” comparable to the identification results using SSU rDNA sequencing or *g6pd* PCR (Table 4), showing that the source of the sample as well as its conservation do not interfere in the HRM protocol. Moreover, the use of HRM protocol is easier than the use of SSU rDNA and/or *g6pd* PCR, since those methods require either sequencing of the product or three or more distinct PCRs followed by gel electrophoresis analysis.

Overall, the *hsp70* HRM protocol described herein accurately and sensitively identified *Leishmania* species that are important in the majority of cases of Leishmaniases in the Brazil and Eurasia. The test is simple and rapid, and its use in the clinic or in research samples has many advantages, such as a lower total cost for the identification of a sample and other characteristics that facilitate its application. There is no need for sequencing or gel fractionation to analyze the product, thus avoiding laboratory contamination with PCR products because these products are discarded without being manipulated. It also reduces the need for trained personnel to analyze the fractionation profile of an electrophoretic gel or sequencing data to provide a result. Also the HRM assay presents a possibility of quantifying parasites present in samples because it is a real-time PCR-based technique. Moreover, the whole process can be automated because the analyzer software will produce the “call” result by comparing the tested samples to the reference sample identities, which must always be included in the reactions.

In conclusion, the protocol described herein is a low cost, reliable, easy to apply, potentially automated procedure that is a good alternative for the detection, quantification and identification of *Leishmania* species in biological and clinical samples.

## Supporting Information

S1 Fig Nucleotide sequence of *hsp70* amplicon 2 and primer localization. The underlined sequences indicate the position of the primers used in real-time PCR assays; the grey boxed nucleotides represent the mutation points found among reference strains of *Leishmania*. The black boxed nucleotides represent mismatches that prevent the annealing of oligonucleotide *hsp70*F1 to the *L*. *(Leishmania)* spp. complementary sequence.
(TIFF)

S2 Fig Electrophoretic profile of conventional PCR products of polymorphic regions of the *hsp70* gene from reference *Leishmania* species. *hsp70* amplicons 1 (A) and *hsp70* amplicons 2 (B) were fractioned by electrophoresis in 1.5% agarose gel and stained with ethidium bromide. DNA from reference strains of *Leishmania* are named as follows: (*Lt*): *L*. *(L*.*) tropica*; (*Ld*): *L*. *(L*.*) donovani*; (*Lc*): *L*. *(L*.*) infantum chagasi*; (*Lj*): *L*. *(L*.*) major*; (*La*): *L*. *(L*.*) amazonensis*; *(Lx)*: *L*. *(L*.*) mexicana;* (*Ll*): *L*. *(V*.*) lainsoni*; (*Lb*): *L*. *(V*.*) braziliensis*; (*Lg*): *L*. *(V*.*) guyanensis*; (*Ln*): *L*. *(V*.*) naiffi* and (*Ls*): *L*. *(V*.*) shawi*. (L): 100 bp DNA ladder and (ntc): no template control, without DNA.
(TIFF)

S3 Fig Efficiency of *hsp70* amplicon 1 real-time PCR for DNA from distinct *Leishmania* species. Standard curves were constructed with recombinant plasmids containing amplicon 1 sequence from *L*. *(L*.*) amazonensis* (A), *L*. *(L*.*) infantum chagasi* (B), *L*. *(V*.*) guyanensis* (C) and *L*. *(V*.*) lainsoni* (D). The assays used as a template underwent a 10-fold serial dilution representing 1 x 10^6^ to 1 x 10^1^ plasmid copies per reaction and were performed in duplicate.
(TIFF)

S4 Fig HRM plots of *hsp70* amplicon 1 from *Trypanosoma*. Representative melting profiles of *hsp70* amplicon 1 obtained from the genomic DNA of *T*. *cruzi* and *T*. *brucei*. (A): Normalized melting curves; (B): normalized difference curves and (C): dispersion graph of individual plots from *T*. *cruzi* (*Tc*) and *T*. *brucei* (*Tb*) compared to *L*. *(L*.*) tropica* (*Lt*).
(TIFF)

S1 Table Polymorphisms detection by *in silico* analysis of *Leishmania hsp70* sequences. The hsp70 regions compassing amplicon 1 or amplicon 2 were retrieved from GenBank Database [32] using the sentence “heat shock protein 70 kDa” as descriptor words in “search” field. The obtained sequences were formatted as FASTA files and aligned on BioEdit Sequence Alignment Editor v.7.1.8 [33]. The identity indexes were obtained by pairwise alignments on BioEdit software. Only sequences encompassing the whole amplicon were analyzed. Theoretical melting temperatures of hypothetic amplicons were calculated using OligoCalc oligonucleotide properties on-line calculator [34].
(DOCX)
