# Supplementary figures and images for: High Resolution Melting Analysis Targeting hsp70 as a Fast and Efficient Method for the Discrimination of Leishmania Species
Source: PLoS Negl Trop Dis. 2016 Feb 29;10(2):e0004485. doi: 10.1371/journal.pntd.0004485 (PMC4771719; doi:10.1371/journal.pntd.0004485)

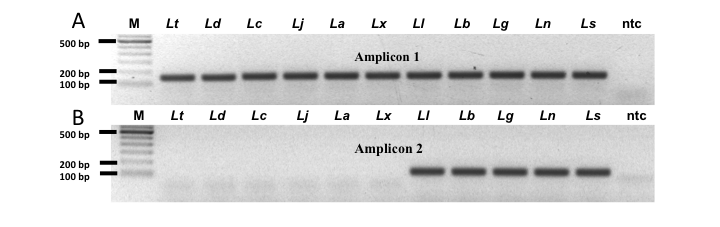

Supplement: S2 Fig — hsp70 amplicons 1 (A) and hsp70 amplicons 2 (B) were fractioned by electrophoresis in 1.5% agarose gel and stained with ethidium bromide. DNA from reference strains of Leishmania are named as follows: (Lt): L. (L.) tropica; (Ld): L. (L.) donovani; (Lc): L. (L.) infantum chagasi; (Lj): L. (L.) major; (La): L. (L.) amazonensis; (Lx): L. (L.) mexicana; (Ll): L. (V.) lainsoni; (Lb): L. (V.) braziliensis; (Lg): L. (V.) guyanensis; (Ln): L. (V.) naiffi and (Ls): L. (V.) shawi. (L): 100 bp DNA ladder and (ntc): no template control, without DNA. (TIFF) [file pntd.0004485.s002.tiff]

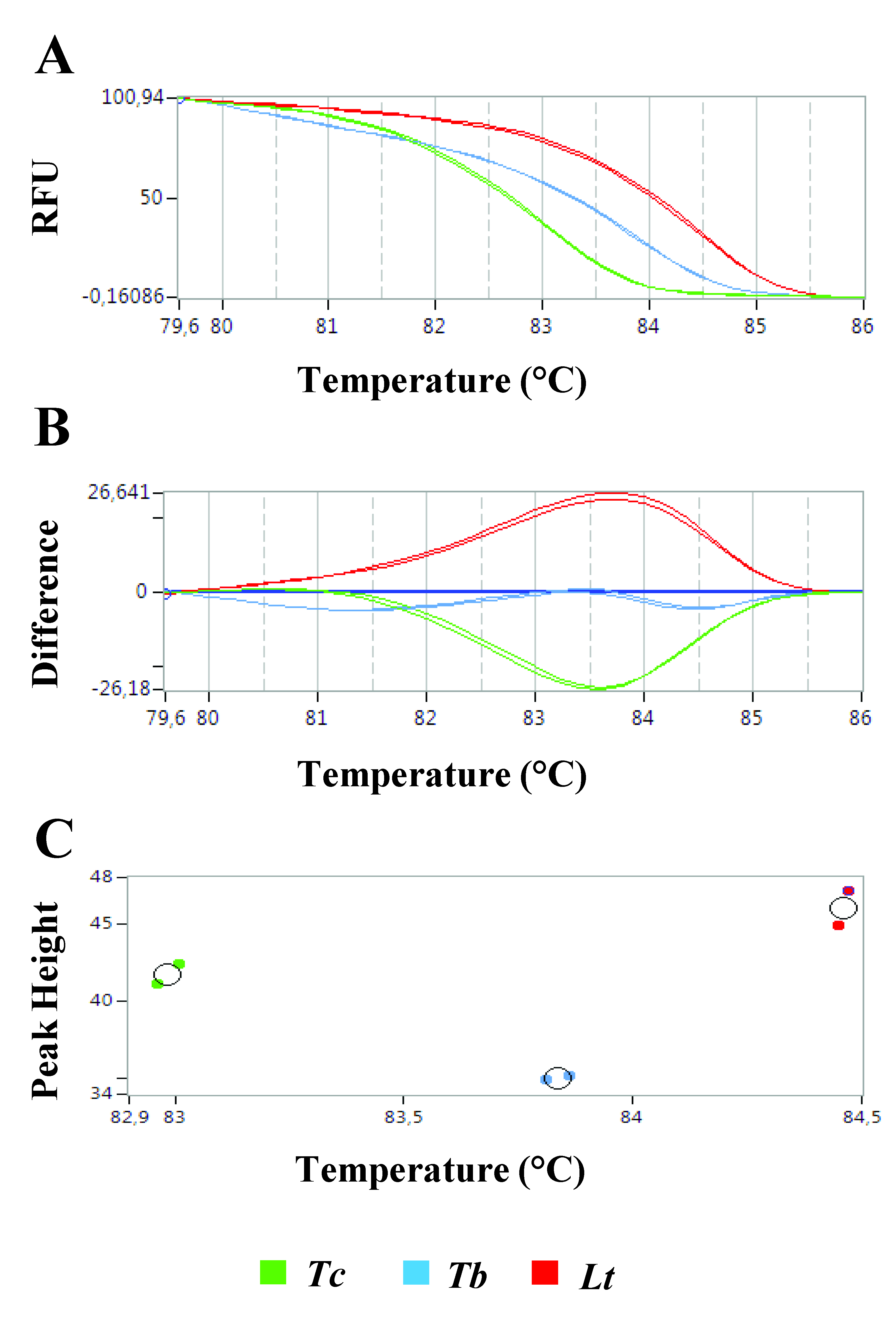

Supplement: S4 Fig — Representative melting profiles of hsp70 amplicon 1 obtained from the genomic DNA of T. cruzi and T. brucei. (A): Normalized melting curves; (B): normalized difference curves and (C): dispersion graph of individual plots from T. cruzi (Tc) and T. brucei (Tb) compared to L. (L.) tropica (Lt). (TIFF) [file pntd.0004485.s004.tiff]
