# Supplementary material for: High Resolution Melting Analysis Targeting hsp70 as a Fast and Efficient Method for the Discrimination of Leishmania Species
Source: PLoS Negl Trop Dis. 2016 Feb 29;10(2):e0004485. doi: 10.1371/journal.pntd.0004485 (PMC4771719; doi:10.1371/journal.pntd.0004485)
Supplement: S1 Table — The hsp70 regions compassing amplicon 1 or amplicon 2 were retrieved from GenBank Database [32] using the sentence “heat shock protein 70 kDa” as descriptor words in “search” field. The obtained sequences were formatted as FASTA files and aligned on BioEdit Sequence Alignment Editor v.7.1.8 [33]. The identity indexes were obtained by pairwise alignments on BioEdit software. Only sequences encompassing the whole amplicon were analyzed. Theoretical melting temperatures of hypothetic amplicons were calculated using OligoCalc oligonucleotide properties on-line calculator [34]. (DOCX) [file pntd.0004485.s005.docx]

**S1 Table – *In silico* analysis of polymorphisms in *hsp70* amplicons 1 and 2.** The nucleotide sequences encompassing *hsp70* amplicons 1 and 2 were retrieved in GenBank database. The sequences were aligned and compared with the reference strains sequences determined in this communication (Fig 1) that are not deposited in the bank (a) or in the absence of this, the comparison was done among the deposited sequences of each species (b); incomplete sequences from GenBank (c); not applicable, (N/A).

| Species | Strain/Isolate Information | Accession # | Amplicon 1 | | Amplicon 2 | |
| --- | --- | --- | --- | --- | --- | --- |
|  |  |  | **Identity Index** | **Theoretical Tm (°C)** | **Identity Index** | **Theoretical Tm (°C)** |
| *L. (L.) tropica* | MHOM/SU/60/OD | a | N/A | 84.1 | N/A | N/A |
| *L. (L.) tropica* | MHOM/IN/79/DD7 | FN395025.1 | 1.000 | 84.1 | N/A | N/A |
| *L. (L.) tropica* | isolate Cat_96 Turkey | KT211405.1 | 1.000 | 84.1 | N/A | N/A |
| *L. (L.) tropica* | isolate Cat_90 Turkey | KT211404.1 | 1.000 | 84.1 | N/A | N/A |
| *L. (L.) tropica* | isolate Cat_89 Turkey | KT211403.1 | 1.000 | 84.1 | N/A | N/A |
| *L. (L.) tropica* | MHOM/EG/90/LPN65 | HF586405.1 | 1.000 | 84.1 | N/A | N/A |
| *L. (L.) tropica* | MHOM/MA/88/LEM1314 | HF586347.1 | 1.000 | 84.1 | N/A | N/A |
| *L. (L.) tropica* | MHOM/KE/81/NLB_030B | FN395026.1 | 0.993 | 84.4 | N/A | N/A |
| *L. (L.) tropica* | MHOM/YE/86/LEM1015 | HF586348.1 | 0.993 | 84.4 | N/A | N/A |
| *L. (L.) donovani* | MHOM/IN/80/DD8 | a | N/A | 84.7 | N/A | N/A |
| *L. (L.) donovani* | LEM138 | JQ990221.1 | 1.000 | 84.7 | N/A | N/A |
| *L. (L.) donovani* | MHOM/IN/1983/AG83 | AY702003.1 | 1.000 | 84.7 | N/A | N/A |
| *L. (L.) donovani* | MHOM/IN/00/DEVI | FN395028.1 | 1.000 | 84.7 | N/A | N/A |
| *L. (L.) donovani* | MHOM/SD/82/Gilani | FN395029.1 | 1.000 | 84.7 | N/A | N/A |
| *L. (L.) donovani* | MHOM/SD/68/1S | FN395027.1 | 1.000 | 84.7 | N/A | N/A |
| *L. (L.) donovani* | voucher VCRC7 India | KF562071.1 | 1.000 | 84.7 | N/A | N/A |
| *L. (L.) donovani* | voucher VCRC4 India | KF562070.1 | 1.000 | 84.7 | N/A | N/A |
| *L. (L.) donovani* | voucher VCRC3 | KF562069.1 | 1.000 | 84.7 | N/A | N/A |
| *L. (L.) donovani* | voucher VCRC2 | KF562068.1 | 1.000 | 84.7 | N/A | N/A |
| *L. (L.) donovani* | IN/VCRC5 | KC884001.1 | 1.000 | 84.7 | N/A | N/A |
| *L. (L.) donovani* | Samtse1 Bhutan | JQ730000.1 | 1.000 | 84.7 | N/A | N/A |
| *L. (L.) donovani* | Trashigang1 Bhutan | JQ729999.1 | 1.000 | 84.7 | N/A | N/A |
| *L. (L.) donovani* | MHOM/IN/1983/AG83 | AY702003.1 | 1.000 | 84.7 | N/A | N/A |
| *L. (L.) donovani* | MHOM/MA/95/CRE72 | HF586352.1 | 1.000 | 84.7 | N/A | N/A |
| *L. (L.) donovani* | DD8 | AY913842.2 | 0.944 | 85.0 | N/A | N/A |
| *L. (L.) infantum chagasi* | MCER/BR/1981/M6445 | a | N/A | 84.7 | N/A | N/A |
| *L. (L.) infantum chagasi* | MHOM/BR/07/ARL | FN395037.1 | 1.000 | 84.7 | N/A | N/A |
| *L. (L.) infantum chagasi* | MHOM/BR/07/WC | FN395036.1 | 1.000 | 84.7 | N/A | N/A |
| *L. (L.) infantum chagasi* | MCAN/BR/06/MAIKE | FN395035.1 | 1.000 | 84.7 | N/A | N/A |
| *L. (L.) infantum* | MHOM/MA/67/IMT-AP263 | FN395033.1 | 1.000 | 84.7 | N/A | N/A |
| *L. (L.) infantum* | MHOM/PT/00/IMT260 | FN395032.1 | 1.000 | 84.7 | N/A | N/A |
| *L. (L.) infantum* | MHOM/MT/85/Buck | FN395031.1 | 1.000 | 84.7 | N/A | N/A |
| *L. (L.) infantum* | isolate 8. Phlebotomus kandelakii. Georgia | JN676923.1 | 1.000 | 84.7 | N/A | N/A |
| *L. (L.) infantum* | isolate 2. Phlebotomus kandelakii. Georgia | JN676922.1 | 1.000 | 84.7 | N/A | N/A |
| *L. (L.) infantum* | isolate 4. Phlebotomus balcanicus. Georgia | JN676921.1 | 1.000 | 84.7 | N/A | N/A |
| *L. (L.) infantum* | MHOM/ES/90/BCN61 | HF586351.1 | 1.000 | 84.7 | N/A | N/A |
| *L. (L.) infantum* | MHOM/DZ/94/CRE64 | HF586349.1 | 1.000 | 84.7 | N/A | N/A |
| *L. (L.) infantum* | LEM75 /zymodeme 1 | X85798.1 | 0.972 | 84.1 | N/A | N/A |
| *L. (L.) infantum* | MCAN/IR/96/LON-49 | GQ121006.1 | 0.875 | 85.3 | N/A | N/A |
| *L. (L.) major* | MHOM/IL/81/Friedlin | a | N/A | 85.0 | N/A | N/A |
| *L. (L.) major* | strain L137. Spain | FN395023.1 | 1.000 | 85.0 | N/A | N/A |
| *L. (L.) major* | strain Githure. Kenya | FN395024.1 | 1.000 | 85.0 | N/A | N/A |
| *L. (L.) major* | strain UQ_8. Sudan | FN395022.1 | 1.000 | 85.0 | N/A | N/A |
| *L. (L.) major* | isolate Cat_74. blood. Turkey | KT211401.1 | 1.000 | 85.0 | N/A | N/A |
| *L. (L.) major* | isolate Cat_38. blood. Turkey | KT211400.1 | 1.000 | 85.0 | N/A | N/A |
| *L. (L.) major* | isolate Cat_30. blood. Turkey | KT211399.1 | 1.000 | 85.0 | N/A | N/A |
| *L. (L.) major* | MHOM/SD/2003/LCB33 | HF586346.1 | 1.000 | 85.0 | N/A | N/A |
| *L. (L.) major* | MHOM/BF/2004/REN04-8 | HF586345.1 | 1.000 | 85.0 | N/A | N/A |
| *L. (L.) amazonensis* | MHOM/BR/1973/M2269 | a | N/A | 85.0 | N/A | N/A |
| *L. (L.) amazonensis* | MHOM/BR/73/M2269 | EU599090.1 | 1.000 | 85.0 | N/A | N/A |
| *L. (L.) amazonensis* | MHOM/BR/77/LTB0016/C1S1 | L14604.1 | 1.000 | 85.0 | N/A | N/A |
| *L. (L.) amazonensis* | MHOM/BR/77/LTB0016/C1S1 | L14605.1 | 1.000 | 85.0 | N/A | N/A |
| *L. (L.) amazonensis* | MHOM/CO/82/CELIS | HF586353.1 | 1.000 | 85.0 | N/A | N/A |
| *L. (L.) mexicana* | MNYC/BZ/62/M379 | a | N/A | 85.0 | N/A | N/A |
| *L. (L.) mexicana* | MNYC/BZ/62/M379 | EU599091.1 | 1.000 | 85.0 | N/A | N/A |
| *L. (L.) mexicana* | MHOM/PE/02/LH2312 | FN395038.1 | 0.986 | 85.0 | N/A | N/A |
| *L. (V.) lainsoni* | MHOM/BR/81/M6426 | a | N/A | 85.0 | N/A | 82.9 |
| *L. (V.) lainsoni* | MHOM/BR/1981/M6426 | GU071174.1 | 1.000 | 85.0 | 1.000 | 82.9 |
| *L. (V.) lainsoni* | MAGO/BR/1983/IM1721 | GU071176.1 | 1.000 | 85.0 | 1.000 | 82.9 |
| *L. (V.) lainsoni* | MHOM/PE/02/LH2344 | FN395049.2 | 0.979 | 85.0 | 1.000 | 82.9 |
| *L. (V.) lainsoni* | MHOM/BR/2002/RBO027-P | GU071179.1 | 0.979 | 85.0 | 1.000 | 82.9 |
| *L. (V.) lainsoni* | MHOM/PE/03/LC2525 | FN395050.1 | 0.979 | 85.0 | 1.000 | 82.9 |
| *L. (V.) lainsoni* | MHOM/PE/91/LC1581 | FN395048.1 | 0.979 | 85.0 | 1.000 | 82.9 |
| *L. (V.) lainsoni* | MHOM/BO/95/CUM71 | FN395047.1 | 0.979 | 85.0 | 1.000 | 82.9 |
| *L. (V.) lainsoni* | MHOM/BR/2002/NMT-RBO004 | GU071187.1 | 0.972 | 85.3 | 1.000 | 82.9 |
| *L. (V.) lainsoni* | MCOE/BR/1983/IM1367 | GU071182.1 | 0.993 | 85.3 | 1.000 | 82.9 |
| *L. (V.) braziliensis* | MHOM/BR/1975/M2903 | a | N/A | 85.6 | N/A | 83.3 |
| *L. (V.) braziliensis* | MHOM/BR/1975/M2903 | GU071173.1 | 1.000 | 85.6 | 1.000 | 83.3 |
| *L. (V.) braziliensis* | MHOM/BR/M2903 | M87878.1 | 1.000 | 85.6 | 1.000 | 83.3 |
| *L. (V.) braziliensis* | MHOM/BR/2002/NMT-LTCP 14621-P clone A | GU368195.1 | 1.000 | 85.6 | 1.000 | 83.3 |
| *L. (V.) braziliensis* | MHOM/BR/2002/NMT-LTCP 14418-P clone B | GU368205.1 | 1.000 | 85.6 | 1.000 | 83.3 |
| *L. (V.) braziliensis* | MHOM/BR/2001/NMT-LTCP14369-P clone B | GU368209.1 | 1.000 | 85.6 | 1.000 | 83.3 |
| *L. (V.) braziliensis* | MHOM/BR/2002/NMT-LTCP 14616-P clone C | GU368194.1 | 1.000 | 85.6 | 1.000 | 83.3 |
| *L. (V.) braziliensis* | MHOM/BR/2001/RGJ clone B | GU368182.1 | 1.000 | 85.6 | 1.000 | 83.3 |
| *L. (V.) braziliensis* | MHOM/BR/2002/NMT-LTCP14566-P | GU368202.1 | 1.000 | 85.6 | 1.000 | 83.3 |
| *L. (V.) braziliensis* | MHOM/BR/2006/ICA | GU368201.1 | 1.000 | 85.6 | 1.000 | 83.3 |
| *L. (V.) braziliensis* | MHOM/BR/2002/NMT-LTCP 14627-P clone B | GU368200.1 | 1.000 | 85.6 | 1.000 | 83.3 |
| *L. (V.) braziliensis* | MHOM/BR/2001/RGJ clone A | GU368181.1 | 1.000 | 85.6 | 1.000 | 83.3 |
| *L. (V.) braziliensis* | MHOM/BR/2002/NMT-LTCP 14624-P clone B | GU368198.1 | 1.000 | 85.6 | 1.000 | 83.3 |
| *L. (V.) braziliensis* | MHOM/BR/2002/EMM clone B | GU368180.1 | 1.000 | 85.6 | 1.000 | 83.3 |
| *L. (V.) braziliensis* | MHOM/PE/02/LH2182 | FN395040.1 | 1.000 | 85.6 | 1.000 | 83.3 |
| *L. (V.) braziliensis* | MHOM/BR/2002/NMT-LTCP 14440-P clone B | GU368187.1 | 1.000 | 85.6 | 1.000 | 83.3 |
| *L. (V.) braziliensis* | MHOM/BR/2002/NMT-LTCP 14440-P clone A | GU368186.1 | 1.000 | 85.6 | 1.000 | 83.3 |
| *L. (V.) braziliensis* | MHOM/BR/2002/LTCP14432 | GU368185.1 | 1.000 | 85.6 | 1.000 | 83.3 |
| *L. (V.) braziliensis* | MHOM/BR/2002/NMT-LTCP 14515-P | GU368191.1 | 1.000 | 85.6 | 1.000 | 83.3 |
| *L. (V.) braziliensis* | MHOM/BR/2002/NMT-LTCP 14451-P | GU368190.1 | 1.000 | 85.6 | 1.000 | 83.3 |
| *L. (V.) braziliensis* | MHOM/BR/2002/NMT-LTCP 14447-P clone B | GU368189.1 | 1.000 | 85.6 | 1.000 | 83.3 |
| *L. (V.) braziliensis* | MHOM/BR/2002/LTCP14438 | GU368184.1 | 1.000 | 85.6 | 1.000 | 83.3 |
| *L. (V.) braziliensis* | MHOM/BR/06/ICA | FN395043.1 | 1.000 | 85.6 | 1.000 | 83.3 |
| *L. (V.) braziliensis* | MHOM/BR/2002/EMM clone A | GU368179.1 | 1.000 | 85.6 | 0.990 | 82.9 |
| *L. (V.) braziliensis* | MHOM/PE/91/LC2177CL2 | EU599088.1 | 0.993 | 85.9 | 1.000 | 83.3 |
| *L. (V.) braziliensis* | MHOM/BR/2002/NMT-LTCP14563-P | GU368210.1 | 0.993 | 85.9 | 1.000 | 83.3 |
| *L. (V.) braziliensis* | MHOM/BR/2001/NMT-LTCP14369-P clone A | GU368208.1 | 0.993 | 85.9 | 1.000 | 83.3 |
| *L. (V.) braziliensis* | MHOM/BR/2002/NMT-LTCP 14509-P clone B | GU368207.1 | 0.993 | 85.9 | 1.000 | 83.3 |
| *L. (V.) braziliensis* | MHOM/BR/2002/NMT-LTCP 14509-P clone A | GU368206.1 | 0.993 | 85.9 | 1.000 | 83.3 |
| *L. (V.) braziliensis* | MHOM/BR/2002/NMT-LTCP 14418-P clone A | GU368204.1 | 0.993 | 85.9 | 1.000 | 83.3 |
| *L. (V.) braziliensis* | MHOM/BR/2001/JOLIVAL | GU368203.1 | 0.993 | 85.9 | 1.000 | 83.3 |
| *L. (V.) braziliensis* | MHOM/BR/2002/NMT-LTCP 14627-P clone A | GU368199.1 | 0.993 | 85.9 | 1.000 | 83.3 |
| *L. (V.) braziliensis* | MHOM/BR/2002/NMT-LTCP 14624-P clone A | GU368197.1 | 0.993 | 85.9 | 1.000 | 83.3 |
| *L. (V.) braziliensis* | MHOM/BR/2002/NMT-LTCP 14621-P clone B | GU368196.1 | 0.993 | 85.9 | 1.000 | 83.3 |
| *L. (V.) braziliensis* | MHOM/BR/2002/NMT-LTCP 14616-P clone B | GU368193.1 | 0.993 | 85.9 | 1.000 | 83.3 |
| *L. (V.) braziliensis* | MHOM/BR/2002/NMT-LTCP 14447-P clone A | GU368188.1 | 0.993 | 85.9 | 1.000 | 83.3 |
| *L. (V.) braziliensis* | MHOM/BR/2001/RGJ clone C | GU368183.1 | 0.993 | 85.9 | 1.000 | 83.3 |
| *L. (V.) braziliensis* | MHOM/PE/91/LC2177 | FN395042.1 | 0.993 | 85.9 | 1.000 | 83.3 |
| *L. (V.) braziliensis* | MHOM/BO/94/CUM29 | FN395041.1 | 0.993 | 85.9 | 1.000 | 83.3 |
| *L. (V.) braziliensis* | MHOM/BO/--/CUM180 | FN395039.1 | 0.993 | 85.9 | 1.000 | 83.3 |
| *L. (V.) braziliensis* | MHOM/BR/2000/LTCP1339 | GU071186.1 | 0.993 | 85.9 | 1.000 | 83.3 |
| *L. (V.) braziliensis* | MHOM/PE/90/FY | HF586372.1 | 0.993 | 85.9 | 1.000 | 83.3 |
| *L. (V.) braziliensis* | MHOM/CO/90/LEM2216 | HF586371.1 | 0.993 | 85.9 | 1.000 | 83.3 |
| *L. (V.) braziliensis* | MHOM/BR/2002/LMG | GU071181.1 | 0.986 | 85.6 | 1.000 | 83.3 |
| *L. (V.) braziliensis* | MHOM/BO/90/AN | HF586370.1 | c | c | 1.000 | 83.3 |
| *L. (V.) braziliensis* | IWHI/BR/86/M10187 | HF586369.1 | c | c | 1.000 | 83.3 |
| *L. (V.) braziliensis* | MHOM/PE/--/LH1099 | HF586375.1 | c | c | 1.000 | 83.3 |
| *L. (V.) braziliensis* | MHOM/BR/00/LTB333 | HF586404.1 | c | c | 1.000 | 83.3 |
| *L. (V.) braziliensis* | MHOM/BR/2002/NMT-RBO037 | GU071180.1 | 0.986 | 85.9 | 0.962 | 83.3 |
| *L. (V.) braziliensis* | MHOM/BR/2002/NMT-LTCP 14616-P clone A | GU368192.1 | 0.986 | 86.1 | 0.990 | 83.7 |
| *L. (V.) braziliensis* | Boytron | X62485.1 | 0.986 | 85.6 | 0.952 | 82.9 |
| *L. (V.) guyanensis* | MHOM/BR/1975/M4147 | a | N/A | 85.6 | N/A | 82.9 |
| *L. (V.) guyanensis* | MHOM/BR/75/M4177 | EU599093.1 | 1.000 | 85.6 | 1.000 | 82.9 |
| *L. (V.) guyanensis* | MHOM/BR/1997/NMT-MAO 317P | GU368240.1 | 1.000 | 85.6 | 1.000 | 82.9 |
| *L. (V.) guyanensis* | MHOM/BR/1997/NMT-MAO 316P | GU368239.1 | 1.000 | 85.6 | 1.000 | 82.9 |
| *L. (V.) guyanensis* | MHOM/BR/1997/NMT-MAO 299P | GU368238.1 | 1.000 | 85.6 | 1.000 | 82.9 |
| *L. (V.) guyanensis* | MHOM/BR/1997/NMT-MAO 292G | GU368237.1 | 1.000 | 85.6 | 1.000 | 82.9 |
| *L. (V.) guyanensis* | MHOM/BR/1997/NMT-MAO 292P | GU368236.1 | 1.000 | 85.6 | 1.000 | 82.9 |
| *L. (V.) guyanensis* | MHOM/BR/1997/NMT-MAO 258P | GU368235.1 | 1.000 | 85.6 | 1.000 | 82.9 |
| *L. (V.) guyanensis* | MHOM/BR/1997/NMT-MAO 253P | GU368234.1 | 1.000 | 85.6 | 1.000 | 82.9 |
| *L. (V.) guyanensis* | MHOM/BR/1997/NMT-MAO 248P | GU368233.1 | 1.000 | 85.6 | 1.000 | 82.9 |
| *L. (V.) guyanensis* | MHOM/BR/1997/NMT-MAO 247P | GU368232.1 | 1.000 | 85.6 | 1.000 | 82.9 |
| *L. (V.) guyanensis* | MHOM/BR/1997/NMT-MAO 246P | GU368231.1 | 1.000 | 85.6 | 1.000 | 82.9 |
| *L. (V.) guyanensis* | MHOM/BR/1997/NMT-MAO 243P | GU368230.1 | 1.000 | 85.6 | 1.000 | 82.9 |
| *L. (V.) guyanensis* | MHOM/BR/1997/NMT-MAO 233P | GU368229.1 | 1.000 | 85.6 | 1.000 | 82.9 |
| *L. (V.) guyanensis* | MHOM/BR/1997/NMT-MAO 223P | GU368228.1 | 1.000 | 85.6 | 1.000 | 82.9 |
| *L. (V.) guyanensis* | MHOM/BR/1997/NMT-MAO 202P | GU368227.1 | 1.000 | 85.6 | 1.000 | 82.9 |
| *L. (V.) guyanensis* | MHOM/BR/2007/029-ZAV | GU368226.1 | 1.000 | 85.6 | 1.000 | 82.9 |
| *L. (V.) guyanensis* | MHOM/BR/2007/025-LFA | GU368225.1 | 1.000 | 85.6 | 1.000 | 82.9 |
| *L. (V.) guyanensis* | MHOM/BR/2007/018-MAS | GU368224.1 | 1.000 | 85.6 | 1.000 | 82.9 |
| *L. (V.) guyanensis* | MHOM/BR/2007/014-JIS | GU368223.1 | 1.000 | 85.6 | 1.000 | 82.9 |
| *L. (V.) guyanensis* | MHOM/BR/2007/011 | GU368222.1 | 1.000 | 85.6 | 1.000 | 82.9 |
| *L. (V.) guyanensis* | MHOM/BR/1997/NMT-MAO 342P | GU368221.1 | 1.000 | 85.6 | 1.000 | 82.9 |
| *L. (V.) guyanensis* | MHOM/BR/1997/NMT-MAO 328P | GU368220.1 | 1.000 | 85.6 | 1.000 | 82.9 |
| *L. (V.) guyanensis* | MHOM/BR/1997/NMT-MAO 325P | GU368219.1 | 1.000 | 85.6 | 1.000 | 82.9 |
| *L. (V.) guyanensis* | MHOM/BR/1997/NMT-MAO 324P | GU368218.1 | 1.000 | 85.6 | 1.000 | 82.9 |
| *L. (V.) guyanensis* | MHOM/BR/1997/NMT-MAO 308G | GU368217.1 | 1.000 | 85.6 | 1.000 | 82.9 |
| *L. (V.) guyanensis* | MHOM/BR/1997/NMT-MAO 307P | GU368216.1 | 1.000 | 85.6 | 1.000 | 82.9 |
| *L. (V.) guyanensis* | MHOM/BR/1997/NMT-MAO 264P | GU368215.1 | 1.000 | 85.6 | 1.000 | 82.9 |
| *L. (V.) guyanensis* | MHOM/BR/1997/NMT-MAO 210P | GU368214.1 | 1.000 | 85.6 | 1.000 | 82.9 |
| *L. (V.) guyanensis* | MHOM/BR/1997/NMT-MAO 203G clone A | GU368212.1 | 1.000 | 85.6 | 1.000 | 82.9 |
| *L. (V.) guyanensis* | MHOM/BR/1975/M4147 | GU071172.1 | 1.000 | 85.6 | 1.000 | 82.9 |
| *L. (V.) guyanensis* | MHOM/GF/79/LEM85 | HF586362.1 | 1.000 | 85.6 | 1.000 | 82.9 |
| *L. (V.) guyanensis* | MHOM/BR/1997/NMT-MAO 203P | GU368211.1 | 1.000 | 85.6 | 1.000 | 82.9 |
| *L. (V.) guyanensis* | MHOM/GF/2004/LBC43 | HF586361.1 | 1.000 | 85.6 | 1.000 | 82.9 |
| *L. (V.) guyanensis* | MHOM/BR/07/029-ZAV | FN395053.1 | 1.000 | 85.6 | 1.000 | 82.9 |
| *L. (V.) guyanensis* | strain MHOM/GF/85/LEM699 | FN395052.1 | 1.000 | 85.6 | 1.000 | 82.9 |
| *L. (V.) guyanensis* | MHOM/BR/1997/NMT-MAO 203G clone B | GU368213.1 | 1.000 | 86.6 | 1.000 | 82.9 |
| *L. (V.) guyanensis* | MHOM/CO/83/REST417 | HF586357.1 | 0.993 | 85.9 | 1.000 | 82.9 |
| *L. (V.) guyanensis* | MHOM/PE/02/LH2372 | FN395051.1 | 0.993 | 85.3 | 1.000 | 82.9 |
| *L. (V.) guyanensis* | MHOM/BR/2002/RBO01 | GU071178.1 | 0.993 | 85.3 | 1.000 | 82.9 |
| *L. (V.) guyanensis* | MHOM/GF/2004/GAE1 | HF586406.1 | c | c | 1.000 | 82.9 |
| *L. (V.) guyanensis* | MHOM/EC/90/JUBERLY | HF586360.1 | c | c | 1.000 | 82.9 |
| *L. (V.) guyanensis* | MHOM/EC/90/UI.031 | HF586378.1 | *c* | c | 1.000 | 82.9 |
| *L. (V.) naiffi* | MDAS/BR/1979/M5533 | a | N/A | 86.1 | N/A | 83.3 |
| *L. (V.) naiffi* | MDAS/BR/1979/M5533 | GU071183.1 | 1.000 | 86.1 | 1.000 | 83.3 |
| *L. (V.) naiffi* | ISQU/BR/1994/IM3936 | GU071185.1 | 0.979 | 86.4 | 1.000 | 83.3 |
| *L. (V.) naiffi* | MHOM/GF/97/CRE88 | HF586373.1 | c | c | 1.000 | 83.3 |
| *L. (V.) naiffi* | MDAS/BR/78/M5210 | FN395056.2 | c | c | 1.000 | 83.3 |
| *L. (V.) naiffi* | MHOM/00/94/CRE58 | HF586374.1 | 0.951 | 85.9 | 0.981 | 84.1 |
| *L. (V.) naiffi* | MHOM/BR/1991/IM3740 | GU071184.1 | 0.958 | 86.1 | 0.981 | 84.1 |
| *L. (V.) shawi* | MCEB/BR/1984/M8408 | a | N/A | 86.1 | N/A | 82.9 |
| *L. (V.) shawi* | MCEB/BR/1984/M8408 | GU071177.1 | 1.000 | 86.1 | 1.000 | 82.9 |
| *L. (V.) shawi* | IWHI/BR/1985/IM2326 | GU071175.1 | 0.993 | 85.9 | 1.000 | 82.9 |
| *L. (V.) panamensis* | MHOM/PA/71/LS94 | EU599094.1 | b | 85.9 | 1.000 | 82.9 |
| *L. (V.) panamensis* | MCHO/PA/00/M4039 | FN395055.1 | 1.000 | 85.9 | 1.000 | 82.9 |
| *L. (V.) panamensis* | MHOM/PA/--/Ps | HF586367.1 | 1.000 | 85.9 | 1.000 | 82.9 |
| *L. (V.) panamensis* | MHOM/PA/--/P7 | HF586366.1 | 1.000 | 85.9 | 1.000 | 82.9 |
| *L. (V.) panamensis* | MHOM/PA/--/P6 | HF586365.1 | 1.000 | 85.9 | 1.000 | 82.9 |
| *L. (V.) panamensis* | MHOM/PA/--/P20 | HF586364.1 | 1.000 | 85.9 | 1.000 | 82.9 |
| *L. (V.) panamensis* | MHOM/PA/--/P1 | HF586363.1 | 1.000 | 85.9 | 1.000 | 82.9 |
| *L. (V.) panamensis* | MHOM/CR/2004/TIM13 | HF586359.1 | 1.000 | 85.9 | 1.000 | 82.9 |
| *L. (V.) peruviana* | MHOM/PE/90/LCA08CL2 | EU599089.1 | b | 85.9 | 1.000 | 83.3 |
| *L. (V.) peruviana* | MHOM/PE/90/LC468 | FN395046.1 | 1.000 | 85.9 | 1.000 | 83.3 |
| *L. (V.) peruviana* | MHOM/PE/03/LH2439 | FN395045.1 | 1.000 | 85.9 | 1.000 | 83.3 |
| *L. (V.) peruviana* | MHOM/PE/03/LH2864 | FN395044.1 | 1.000 | 85.9 | 1.000 | 83.3 |
| *L. (V.) peruviana* | MHOM/PE/89/LH741 | HF586368.1 | 1.000 | 85.9 | 1.000 | 83.3 |
| *L. (L.) aethiopica* | MHOM/ET/72/L100 | FN395021.1 | b | 84.1 | N/A | N/A |
| *L. (L.) aethiopica* | MHOM/ET/83/169-83 | FN395020.1 | 1.000 | 84.1 | N/A | N/A |
| *L. (L.) aethiopica* | NLB_107-08 | FN395019.1 | 1.000 | 84.1 | N/A | N/A |
| *L. (L.) aethiopica* | MHOM/ET/89/GERE | FN395018.1 | 1.000 | 84.1 | N/A | N/A |
| *L. (L.) martiniquensis* | MHOM/TH/2013/LSCM3 | KP244368.1 | b | 84.1 | N/A | N/A |
| *L. (L.) martiniquensis* | MHOM/TH/2013/LSCM2 | KP244367.1 | 1.000 | 84.1 | N/A | N/A |
| *L. (L.) martiniquensis* | MHOM/TH/2012/LSCM1 | KP244366.1 | 1.000 | 84.1 | N/A | N/A |
| *L. (L.) martiniquensis* | MHOM/MQ/1992/MAR1 | KP244365.1 | 1.000 | 84.1 | N/A | N/A |
| *Leishmania 'siamensis'* | isolate CU1 | JX852709.1 | b | 84.1 | N/A | N/A |
| *Leishmania 'siamensis'* | isolate PCM5 | KC202881.1 | 1.000 | 84.1 | N/A | N/A |
| *Leishmania 'siamensis'* | isolate PCM4 | KC202882.1 | 1.000 | 84.1 | N/A | N/A |
| *Leishmania 'siamensis'* | isolate CU1 | KC202883.1 | 1.000 | 84.1 | N/A | N/A |
| *Leishmania 'siamensis'* | isolate 4-2 | JX852708.1 | 0.993 | 83.9 | N/A | N/A |
| *Leishmania 'siamensis'* | isolate PCM2 | KC202880.1 | 0.910 | 83.3 | N/A | N/A |
